# Intraindividual comparison between ^68^Ga-PSMA-PET/CT and mpMRI for intraprostatic tumor delineation in patients with primary prostate cancer: a retrospective analysis in 101 patients

**DOI:** 10.1007/s00259-020-04827-6

**Published:** 2020-04-28

**Authors:** Simon Spohn, Chiara Jaegle, Thomas F. Fassbender, Tanja Sprave, Eleni Gkika, Nils H. Nicolay, Michael Bock, Juri Ruf, Matthias Benndorf, Christian Gratzke, Anca L. Grosu, Constantinos Zamboglou

**Affiliations:** 1 Department of Radiation Oncology, Faculty of Medicine, University of Freiburg, Freiburg im Breisgau, Baden-Württemberg Germany; 2 German Cancer Consortium (DKTK), Partner Site Freiburg, Freiburg, Germany; 3 Department of Nuclear Medicine, Faculty of Medicine, University of Freiburg, Freiburg im Breisgau, Baden-Württemberg Germany; 4 Department of Radiology, Faculty of Medicine, University of Freiburg, Freiburg im Breisgau, Baden-Württemberg Germany; 5 Department of Urology; Faculty of Medicine, University of Freiburg, Freiburg im Breisgau, Baden-Württemberg Germany; 6 Berta-Ottenstein-Programme, Faculty of Medicine, University of Freiburg, Freiburg im Breisgau, Baden-Württemberg Germany

**Keywords:** Prostate cancer, MRI, PSMA PET/CT

## Abstract

**Purpose:**

Accurate delineation of intraprostatic gross tumor volume (GTV) is mandatory for successful fusion biopsy guidance and focal therapy planning of prostate cancer (PCa). Multiparametric magnetic resonance imaging (mpMRI) is the current gold standard for GTV delineation; however, prostate-specific membrane antigen positron emission tomography (PSMA-PET) is emerging as a promising alternative. This study compares GTV delineation between mpMRI and ^68^Ga-PSMA-PET in a large number of patients using validated contouring approaches.

**Methods:**

One hundred one patients with biopsy-proven primary PCa who underwent mpMRI and ^68^Ga-PSMA-PET within 3 months before primary treatment were retrospectively enrolled. Clinical parameters (age, PSA, Gleason score in biopsy) were documented. GTV based on MRI and PET images were delineated; volumes measured and laterality determined. Additionally, biopsy data from 77 patients was analyzed. Univariate and multivariate binary logistic regression analyses were performed using concordance in laterality as the endpoint.

**Results:**

In total mpMRI and ^68^Ga-PSMA-PET detected 151 and 159 lesions, respectively. Median GTV-MRI (2.8 ml, 95% CI 2.31–3.38 ml) was significantly (*p* < 0.0001) smaller than median GTV-PET (4.9 ml, 95% CI 3.9–6.6 ml). ^68^Ga-PSMA-PET detected significantly more bilateral lesions than mpMRI (71 vs 57, *p* = 0.03). Analysis of patients with bilateral lesions in biopsy showed a significant higher concordance of laterality in ^68^Ga-PSMA-PET (*p* = 0.03). In univariate analysis, PSA level and volume of GTV-MRI had an impact on concordance in laterality (*p* = 0.02 and *p* = 0.01), whereas in multivariate analysis, only GTV-MRI volume remained significant (*p* = 0.04).

**Conclusion:**

MpMRI and ^68^Ga-PSMA-PET detect a similar amount of PCa lesions. However, GTV-PET had approximately twice the volume (median 4.9 ml vs 2.8 ml) and detected significantly more bilateral lesions than mpMRI. Thus, ^68^Ga-PSMA-PET gives highly important complementary information. Since we could not find any strong evidence for parameters to guide when ^68^Ga-PSMA-PET is dispensable, it should be performed additionally to MRI in patients with intermediate and high-risk PCa according to D’Amico classification to improve GTV delineation.

## Introduction

Prostate cancer (PCa) is the most common tumor entity for men in North America [1] and Europe [2], and PCa incidence rates are rising steadily in Asian countries as well [3]. The accurate delineation of intraprostatic tumor burden is mandatory for successful fusion biopsy guidance [4] and for focal therapy planning such as focal dose escalation in radiotherapy (RT), high-intensity focused ultrasound (HIFU) focal laser ablation (FAL), cryotherapy, or irreversible electroporation (IRE) [5].

Multiparametric magnetic resonance imaging (mpMRI) is the current gold standard for PCa detection [4] but has been shown to miss pivotal tumor lesions and underestimates their volume [6, 7]. Instead prostate-specific membrane antigen positron emission tomography (PSMA-PET) is emerging as a promising technique to improve tumor lesion detection [8–15], focal therapy guidance [16], and non-invasive PCa characterization [17].

In histopathologic comparison studies combining T2-weighted spin echo (T2) and diffusion-weighted (DWI) and dynamic contrast-enhanced (DCE) MRI, with median sensitivity of 61.5% and median specificity of 85.5%, was found. For PSMA-PET, these studies identified similar specificity scores and slightly higher sensitivity scores with a median sensitivity of 76% [15, 18–25]. Additionally, tumor volumes delineated in PSMA-PET and mpMRI differed significantly, with smaller volumes in mpMRI [24, 25].

However, these studies only included small patient numbers (range, 7–53 patients) and used not validated contouring approaches for delineation of intraprostatic tumor according to PSMA PET. The aim of this study is an intraindividual comparison of tumor volume delineation between mpMRI and ^68^Ga-PSMA-PET in a large number of patients (*n* = 101) using validated contouring approaches for both imaging modalities [16, 26]. We analyzed differences in the number of detected PCa lesions and their gross tumor volume (GTV). Additionally, laterality in imaging and biopsies was assessed on a lobe level. Finally, we investigated whether clinical parameters correlate with the concordance of laterality between mpMRI and ^68^Ga-PSMA-PET.

## Patients and methods

### Study design and patient population

A total of 101 patients with biopsy-proven primary PCa who underwent mpMRI and ^68^Ga-PSMA-PET/CT (^68^Ga-PSMA-PET) within 3 months were retrospectively studied. Seven patients had received a transurethral resection of the prostate (TUR-P) more than 2 years prior to imaging and had visible tumor burden in imaging. Exclusion criteria were any therapeutic interventions prior imaging (except for TUR-*P* > 2 years ago), including neoadjuvant androgen deprivation therapy. Clinical parameters (age, prostate-specific antigen (PSA) serum levels prior to conduction of first imaging method (PSA), Gleason score in biopsy) were acquired. The study was approved by the institutional review board under registration number 203/19.

### MR imaging

Prostate MRI was performed in 50 patients on a clinical 3 T system (MAGNETOM Trio Trim, MAGNETOM Skyra, MAGNETOM Vida, Siemens; Discovery MR750, GE Healthcare) and in 51 patients on a 1.5 T MRI (MAGNETOM Area, MAGNETOM Avanto fit, Siemens). For image acquisition, a surface phased array (body matrix) was combined with integrated spine array coil. Endorectal coils were not used to increase both patient compliance and signal homogeneity over the prostate. As part of the imaging protocol, T2-weighted, diffusion-weighted (DWI), and dynamic contrast-enhanced (DCE) image data was acquired. From the DWI images, maps of the apparent diffusion coefficient (ADC) were calculated. A detailed description of the imaging protocols can be found in [25]. Due to the time period of data (2013–2019) acquisition, MR protocols were heterogeneous in terms of slice thickness (3–3,5 mm), gap between slices (0–≤ 1 mm), and *b* values (low, 0 s/mm^2^ and 50 s/mm^2^; high, 800 s/mm^2^, 1000 s/mm^2^, and 1500s/mm^2^).

### PET/CT imaging

The PSMA-HBED-CC radiolabeling with ^68^GaCl_3_ was performed according to good laboratory practice using a fully automated synthesis module (Ecker & Ziegler, Germany) and sterile single-use cassettes. The decay-corrected yield was > 95%, and radiochemical purity of the final product was ≥ 97%. The mean injected activity of ^68^Ga-HBED-CC-PSMA was 205.4 MBq (95% CI 200.1–210.7 MBq). Patients underwent a whole-body PET scan starting 1 h after injection. Scans were performed with either a 64-slice Gemini TF PET/CT scanner, a 16-slice Gemini TF big bore, or Vereos PET/CT scanner (all Philips Healthcare, USA). Cross-calibration of the three scanners was performed to ensure comparability of the quantitative measurements. At the time of the PET scan, either a contrast-enhanced diagnostic CT scan (120 kVp, 100–400 mAs, dose modulation) or a low-dose CT scan (120 kVp, 25 mAs) was performed for attenuation correction (depending on previous CT scans and contraindications). The uptake of ^68^Ga-PSMA was quantified in terms of standardized uptake values (SUV) normalized body weight.

### Image analysis

MpMRI (T2-w and ADC) and PET (attenuation-corrected)/CT DICOM datasets were imported into a radiation treatment planning system (iPLAN RT image 4.1.2 BrainLAB, Germany; or Eclipse™ Treatment Planning System, Varian, USA). Images were co-registered according to in-house protocols [27].

GTV based on MRI T2-w and ADC images (GTV-MRI) was delineated in consensus by two experienced readers. Standardized imaging criteria (PI-RADSv2) were applied, and only lesions with a PI-RADS score of ≥ 3 were considered as relevant [26]. Access to the full MRI datasets including DCE and DWI images side by side was granted to the delineating physician.

**Fig. 1:**
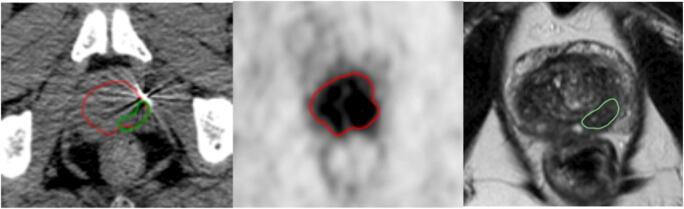
Example of differences in tumor volumes and laterality. (Left) axial CT slice with fused gross tumor volumes (GTV) delineated in PET (GTV-PET, red) and mpMRI (GTV MRI, green). (Middle) GTV delineated in PSMA-PET (scaling SUVmin−max 0–5). (Right) GTV delineated in mpMRI, image shows axial T2-weighted MR image. GTV-PET is larger than GTV delineated in MRI. Additionally, the GTV-PET extents to the right and left lobe, whereas the GTV-MRI is restricted to left lobe only

GTV based on PET images (GTV-PET) was delineated by two experienced readers in consensus. Any monofocal or multifocal uptake greater than the adjacent background uptake in more than one slice within the CT-defined prostate gland was defined as the presence of PCa. GTVs were delineated manually in every single slice using PET image scaling of SUVmin−max: 0–5 [16]. For an example, see Fig. 1.

Subsequently delineated volumes were measured, and laterality (left lobe, right lobe, both lobes) as well as the number of lesions in both modalities was determined. Additionally, laterality of PCa-positive biopsies was extracted from 77 patients. The data was available in a form of histopathological or tumor boards reports.

### Statistical analysis

Statistical analysis was performed using SPSS v25 (IBM, Armonk, NY) for the regression analysis and GraphPad PRISM v7.01 (GraphPad software, San Diego, CA) for all other analyses. D’Agostino-Pearson normality test was performed, and the data was not normally distributed. Comparison of GTV volumes was performed with Wilcoxon matched-pairs signed-rank test. Fisher’s exact test was performed to evaluate concordance in laterality. Univariate and multivariate analysis was performed with binary logistic regression analysis. Concordance in laterality was defined as the endpoint for regression analyses at a statistical significance threshold of < 0.05.

Figures were created with SPSS v25 (IBM, Armonk, NY), GraphPad PRISM v7.01 (GraphPad software, San Diego, CA), and Microsoft Office 2010 (Microsoft, Redmond, WA, USA).

## Results

Between November 2013 and April 2019, 101 patients with primary PCa underwent mpMRI of the pelvis and ^68^Ga-PSMA PET/CT and fulfilled the required inclusion and exclusion criteria. Please see Table 1 for patient characteristics.

**Table 1:** Patient characteristics

patients, *n* = 101	Median (95% CI) or (%)
Age (years)	70 (68–72)
PSA (ng/ml)	10.9 (9.39–13.03)
Patients, *n* = 101	*n* (%)
Gleason score in biopsy, *n*	
6	6 (6)
7a	37 (37)
7b	28 (28)
8	19 (19)
9	11 (11)
D’Amico risk group, *n*	
Low risk	1 (1)
Intermediate risk	35(35)
High risk	65 (65)

### Analysis of GTV, laterality, and biopsies

In MRI, 63 lesions were detected in the left lobe only, 48 lesions in the right lobe only, and 40 lesions in both lobes (in total 151 lesions). In ^68^Ga-PSMA-PET, 51 lesions were detected in the left lobe only, 48 lesions in the right lobe only, and 60 lesions in both lobes (in total 159 lesions).

**Fig. 2:**
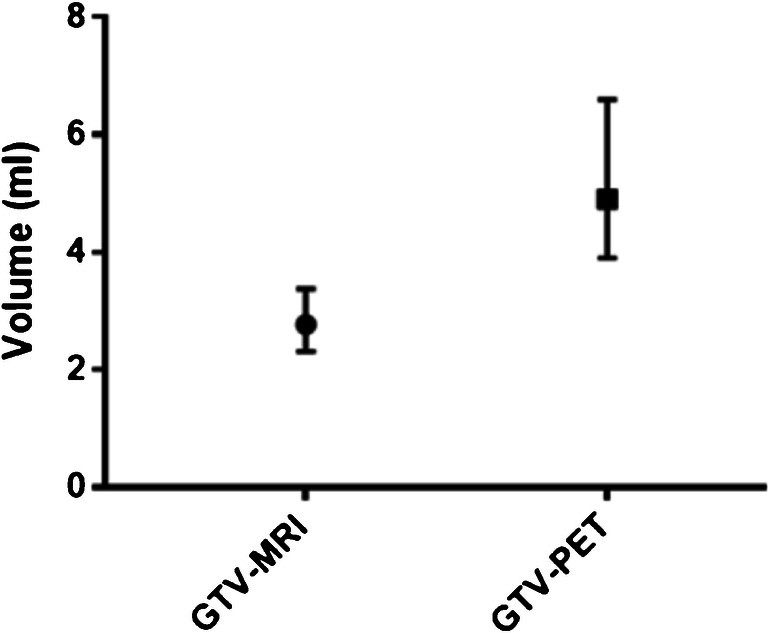
GTV volumes. (Left) median GTV-MRI was 2.8 ml (95% CI 2.31–3.38 ml), and median GTV-PET was 4.9 ml (95% CI 3.9–6.6 ml), respectively. Tumor volume was significantly smaller in mpMRI than in PSMA-PET/CT, *p* < 0.0001

Analysis of tumor volumes revealed that GTV-MRI (median, 2.8 ml; range, 0.1–67.7 ml) was statistically significant smaller than GTV-PET (median 4.9 ml, range 0.5–140.0 ml, *p* < 0.0001). GTVs showed a difference in volume of ≥ 25% in 83.2% of patients and a difference ≥ 50% in 73.3% of patients. For details, see Table 2 and Fig. 2.

**Table 2:** Volume differences

	Volume difference > 25% (in % of patients)	Volume difference > 50% (in % of patients)
GTV-PET>CTV-MRI	62.4%	54.5%
GTV-MRI>GTV-PET	20.8%	19.3%
Total	83.2%	73.8%

**Fig. 3:**
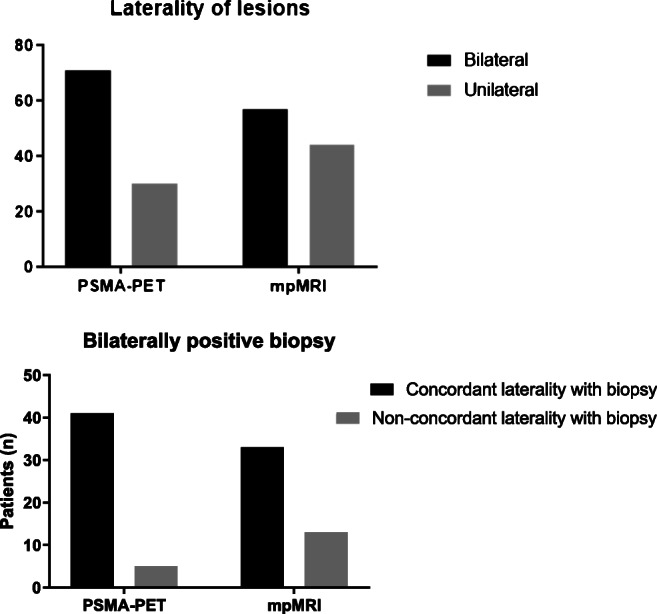
(Upper graph) laterality of lesions: PSMA-PET/CT showed 71 patiens with lesions in both lobes (bilateral) and 30 patients with lesions in one lobe only (unilateral), whereas mpMRI showed 57 patients with lesions in both lobes (bilateral) and 44 patients with lesions in one lobe only (unilateral) lesions. Laterality was significantly different between PSMA-PET/CT and mpMRI, *p* = 0.03. (Lower graph) analysis of biopsy data with bilateral lesions showed 41 concordant and 5 non-concordant cases in PSMA-PET/CT and 33 concordant and 13 non-concordant cases in mpMRI. There is a significant difference between concordance of laterality between PSMA-PET/CT and mpMRI with a higher concordance in PSMA-PET/CT, *p* = 0.03

Distribution and laterality of lesions in one lobe only (unilateral) or both lobes (bilateral) were analyzed. MpMRI showed 44 patients (43.6%) with lesions in one lobe only and 57 patients (56.4%) with lesions in both lobes. ^68^Ga-PSMA-PET showed 30 patients (29.7%) with lesions in one lobe only and 71 patients (70.3%) with lesions in both lobes, respectively (Fig. 3). PSMA-PET detected significantly more bilateral lesions than MRI (*p* = 0.03). In 37.6% of patients, PET and MRI showed different distribution of lesions. PSMA-PET detected lesions in both lobes, whereas MRI did not in 26 patients (26%), and conversely, MRI detected lesions in both lobes, whereas PET did not in 12 patients (12%). Subsequently laterality was concordant in 62.4%.

Biopsy data from histopathological or tumor board reports were available in 77 patients and were analyzed in terms of laterality. Biopsy data showed lesions in one lobe only in 31 patients (40.3%) and lesions on both lobes in 46 patients (59.7%).There is no significant difference between concordance of laterality when analyzing all biopsy data. Subgroup analysis of cases with bilateral lesions in biopsy showed a significant higher concordance of laterality in ^68^Ga-PSMA-PET *p* = 0.03. For details, see Fig. 3.

### Regression analysis

Regression analyses are presented in Table 3. In univariate analysis, PSA level and volume of GTV-MRI had an impact on concordance between ^68^Ga-PSMA-PET and mpMRI in terms of laterality (*p* = 0.02 and *p* = 0.01). Gleason score, MRI technique (1.5 vs 3 T), and age did not show to be significant. In multivariate analysis, including PSA and GTV-MRI volume, only the volume significantly predicted concordance of laterality (*p* = 0.04).

**Table 3:** Regression analysis

	Univariate analysis			Multivariate analysis		
Parameters	OR	95% CI	*p* value	OR	95% CI	*p* value
PSA (metric variable)	0.93	0.88–0.99	0.15	0.96	0.90–1.01	0.14
GTV-MRI (categorical variable)	0.79	0.66–0.94	0.01	0.82	0.69–0.99	0.04
GS (categorical variable)	0.80	0.52–1.23	0.31			
Age (continuous variable)	1.0	0.95–1.05	0.92			
	0.74	0.33–1.65	0.46			

## Discussion

Accurate identification of intraprostatic tumor burden is the key to successful biopsy guidance as well as planning of focal therapies. While mpMRI is the current gold standard, PSMA-PET has been shown to improve detection of intraprostatic tumor lesions. We compared delineation of GTVs in ^68^Ga-PSMA-PET/CT and mpMRI in 101 patients and analyzed clinical and technical parameters in terms of concordance of GTV volumes. Our study included 101 patients and showed that ^68^Ga-PSMA-PET gives complementary information to mpMRI due to identification of larger tumor volumes and more bilateral lesions, which show a higher concordance with biopsies.

mpMRI is the current gold standard for PCa detection [4] and detects significant lesions, since local recurrences occur at the site of primary tumor lesions detected by mpMRI [28, 29]. Nevertheless, Priester et al. demonstrated that mpMRI consistently underestimates the extent of tumor foci [6], and Johnsen et al. showed that mpMRI misses clinically significant tumor lesions in approximately 35% of the patients [7].

A study using whole-mount histopathology as the reference (16) proved that image segmentation based on PET does not overestimate the tumor burden. This study showed that the identified total number of lesions does not differ significantly between ^68^Ga-PSMA-PET and mpMRI. However, GTVs based on MRI were significantly smaller than GTV-PET, with approximately twice the volume in ^68^Ga-PSMA-PET. In more than half of the patients, GTV-PET was ≥ 50% larger than GTV-MRI, and in approximately one fifth of patients GTV-MRI was ≥ 50% larger than GTV-PET. These data confirm that GTVs in mpMRI are smaller than ^68^Ga-PET/CT [24, 25]. These results suggest that the use of margins to the GTV-MRI might deliver a better coverage of the tumor lesions detected by ^68^Ga-PSMA-PET. This approach could be assessed in future studies, which should ideally use whole-mount histology as the reference to draw convincing conclusion.

Additionally, our study showed differences in laterality in imaging modalities with significantly more patients with bilateral lesions in ^68^Ga-PSMA-PET/CT (71 vs 57 patients). Differences in laterality were confirmed by analysis of biopsy data. Comparison with positive bilateral biopsies showed that there is a significantly higher concordance of ^68^Ga-PSMA-PET with biopsy results. A bias in favor of mpMRI is noteworthy, since most biopsies were MRI-guided.

Previous histopathologic comparison studies have shown an increased sensitivity of intraprostatic tumor lesion delineation in small patient numbers. Additionally, the union of GTV-PET and GTV-MRI further improved sensitivity [18, 22, 24]. Consequently, we could prove that there is a significant difference in tumor volume, and laterality in a large number of patients and PET might detect clinically significant tumor lesions in the contralateral prostate lobe, which are not detected by mpMRI. Vice versa mpMRI is not dispensable, due to the substantial number of patients (12%), where mpMRI identified larger volumes and bilateral lesions exclusively. It is important to consider that in this study, contouring in both ^68^Ga-PSMA-PET and MRI was based on guidelines [16, 26]. To our knowledge, this study is the first to compare MRI and ^68^Ga-PET tumor delineation in a large cohort using validated contouring techniques for both imagings. The scaling used for PET results neither in over- nor in underestimation of tumor volumes validated in whole-mount histology reference (16). Our findings show that PSMA-PET gives highly important complimentary information.

Possible reasons for the difference in PCa detection are that mpMRI misses lesions in the transition zone, where conditions such as benign prostatic hypertrophy aggravate identification of tumor lesions [30]. Additionally, artifacts caused by prior biopsies affect mpMRI more than PSMA-PET, since T2-weighted MRI cannot differentiate between PCa lesions and hemorrhage [31]. Plausible biological or physical explanations for differences in volume delineation are not available to our knowledge.

Since the conduction of both imaging techniques in parallel is leading to increased financial burden for the healthcare system and PET-CT causes a radiation exposure, we analyzed the correlation of clinical parameters with the concordance between mpMRI and PET-CT. Thus, we aimed to identify parameters to guide in which cases PET-CT would be dispensable. Univariate analysis showed a significant association for PSA level as well as for volume of GTV-MRI with concordance of laterality between both imaging techniques. In multivariate analysis, only GTV-MRI was significant. Hence, merely in patients with large tumor volumes in mpMRI, ^68^Ga-PSMA-PET/CT might give little complimentary information. However, these patients bear a high risk for lymph node or distant metastases [32], and ^68^Ga-PSMA-PET shows high sensitivities and specificities for lymph node detection, especially in metastases smaller than 10 mm and thus is beneficial for lymph node staging [33, 34]. Consequently, our data encourage the usage of ^68^Ga-PSMA-PET and mpMRI for biopsy guidance and planning of focal therapies and show that ^68^Ga-PSMA-PET should at least be performed in patients with intermediate and high-risk PCa since it gives highly complementary information.

The study’s limitations are the retrospective design and the lack of information about laterality in biopsy samples in 24% of patients. Surely, whole-mount histopathologic data would be the reference gold standard for comparison of intraprostatic tumor burden detection. Conduction of heterogeneous MRI protocols with 1.5 T (without endorectal coil) and 3 T systems is another limitation of our study. However, we performed analyses with data from 50 patients, who received a 3 T mpMRI and obtained comparable results (data not shown). Additionally, we highlight that for the dominant sequences of mpMRI [35], a reasonable and recommended slice thickness of 3 mm was acquired in the vast majority of patients. Furthermore, the inclusion of 1.5 T and 3 T systems reflects real-world prostate diagnostic practice. Potential sources of differences in in visual ^68^Ga-PSMA-PET interpretation were the usage of three different PET-CT scanners and the use of contrast-enhanced as well as low-dose CT-scans. However, cross-calibration between PET-scanners was performed to optimize comparability. ^68^Ga-PSMA accumulation in the bladder intensifies background signal and thus aggravates tumor lesion delineation in areas adjacent to the bladder. New PSMA-tracers like ^18^F-PSMA-1007 show lesser renal elimination and might further improve intraprostatic tumor lesions detection [36, 37].

## Conclusion

Our study is the first study to perform an intraindividual comparison between ^68^Ga-PSMA-PET/CT and mpMRI in a large cohort of > 100 patients by using validated contouring approaches. Taking into account previous findings from histopathologic comparison studies, our study empowers the use of ^68^Ga-PSMA-PET-CT and the combination with mpMRI for delineation of GTVs for planning of focal therapies and biopsy guidance in patients with primary PCa. ^68^Ga-PSMA-PET/CT gives highly important complementary information, which improves coverage of intraprostatic tumor lesions due to identification of larger tumor volumes and bilateral lesions missed by mpMRI. Additionally, ^68^Ga-PSMA-PET detects metastasis with high accuracy and thus is also beneficial for staging. So far there is no strong evidence for parameters to guide in which cases ^68^Ga-PSMA-PET/CT is dispensable.
